# Supplementary material for: Comparative systematics and phylogeography of Quercus Section Cerris in western Eurasia: inferences from plastid and nuclear DNA variation
Source: PeerJ. 2018 Oct 17;6:e5793. doi: 10.7717/peerj.5793 (PMC6195796; doi:10.7717/peerj.5793)
Supplement: File S2 — incuding results of the BLAST identity matches on GenBank. [file peerj-06-5793-s002.docx]

**Supplemental File S2**

**TrnH-psbA Haplotype list, and results of the BLAST identity matches on GenBank**

**Hap_1:** Shared with accession nr. KT963087 (labelled as *Q. baroni*), accession nr. KU240010 (labelled as *Q. dolicholepis*) and accession nr. LM652851 (*Q. spinosa*)^[[1]](#footnote-1)^; 100% identity, no gaps

**142 individuals**

[af01TUN af02TUN af03TUN af04TUN af05TUN af06TUN af07TUN ce01I ce02A ce03A ce04A ce06GR ce07GR ce08GR ce09I ce10I ce11I ce12I ce13I ce14I ce15I ce17TR ce21TR ce22TR ce24TR ce25TR ce26TR ce27TR ce28TR ce29TR ce30TR ce35TR ce37TR ce39HU ce40I ce42A ce44RO ce47I ce48I ce50I ce51RO ce52HR cf01IR cf02IR cr01I cr02I cr03I cr04SLO cr05HR cr06I eu13GR eu14GR eu15GR eu16GR li01TR ml07GR ml08GR ml09GR ml10GR ml11GR ml12GR ml13GR ml14TR ml15TR ml16TR ml17TR ml18TR ml20TR ml21TR ml22TR ml23TR ml26GR su01I su02I su03I su04I su05S su06S su09S su10S su11S su12S su13S su14S su15S su16S su17S su18S su19S su20MO su21MO su22MO su26MO su27MO su28MO su30P su31P su32P su33P su34P su36P su37HR su38I su39I su40F su41ALG su43I su44I su45I su46I su47TUN tj01TR tj02TR tj03TR tj04TR tj05TR tj07TR tj09I tj10I tj11I tj12I tj17GR tj18GR tj19GR tj20GR tj22GR tj23GR tj24I tj25I tj26I tj27I tj28I tj32I tj33TR tj34TR tj35TR tj36TR tj40TR tj41TR tj42TR tj44TR tj45TR]

**Hap_2:** Shared with *Q. coccifera* from N Turkey (Erbaa; LM652816), NW Turkey (Gokceada; LT222166), and NE Greece (Alonyssos; LM652777), *Q. ilex* from Greece (Drymaia; LM652788, Skyathos; LM652791), representatives of the Aegean ‘Cerris-Ilex’ lineage as defined by Simeone et al. (2016); 100% identity, no gaps

**22 individuals**

[ce05GR ce18TR ce23TR ce43SB ce49HR ml01I ml02I ml03I ml04I ml05I ml06I ml24TR ml25TR ml29AL tj06TR tj08I tj21GR tj29I tj30I tj31I tj37TR tj38TR]

**Hap_3:** Shared with *Q*. *libani* (HE591294); 100% identity, no gaps

**2 individuals**

[ce16TR li03TR]

**Hap_4:** NOT SHARED (99% identity with *Q*. *baroni*; LM652806)

**3 individuals**

[ml19TR tj39TR tj43TR]

**Hap_5:** Shared with *Q. coccifera* (LT222169) and *Q. ilex* (LT222196) from S Greece (Crete), representatives of the ‘Cerris-Ilex’ lineage as defined by Simeone et al. (2016); 100% identity, no gaps

**7 individuals**

[br01TR br02TR br03TR br04TR br05TR ce19TR ce20TR]

**Hap_6:** Shared with *Q. coccifera* (LM652772) from S Greece (Crete), representative of the ‘Cerris-Ilex’ lineage as defined by Simeone et al. (2016), and *Q. brantii* (HE591290); 100% identity, no gaps

**6 individuals**

[br06KUR br07IS ce32TR ce33TR ce34TR ml27IS]

**Hap_7:** Shared with *Q. coccifera* (LM652774) from SW Greece (Ikaria), representative of the ‘Cerris-Ilex’ lineage as defined by Simeone et al. (2016); 100% identity, no gaps

**1 individual**

[ce36TR]

**Hap_8:** NOT SHARED (98% identity with *Q. baroni*; LM652806, *Q. pannosa*; LM652829; *Q. pseudosemecarpifolia*; HE591333, *Q. spinosa*; KM841421)

3 Individuals

[li02TR li04TR li05TR]

**Hap_9:** shared with *Q. ithaburensis* (HE591316), 100% identity, no gaps

**10 individuals**

[ce38IS ce45I ce53IS it01IS it02IS it03IS it04IS it05IS lk02IS lk03IS]

**Hap_10**: NOT SHARED (99% identity with *Q*. *coccifera*, LT222169 and *Q*. *ilex*, LT222196, from S Greece - Crete, representatives of the ‘Cerris-Ilex’ lineage as defined by Simeone et al. 2016)

**3 individuals**

[ce41LB ce46I lk01IS]

**Hap_11:** Shared with *Q. ilex* from N Morocco (HE591263) and S France (LM652784), representatives of the ‘West-Med’ lineage in section *Ilex* as defined by Simeone et al. 2016); 100% identity, no gaps

**5 individuals**

[su07S su08S su29P su35P su42S]

**Hap_12:** NOT SHARED (98% identity with *Q. ilex* from N Morocco, HE591263, and S France (LM652784), representatives of the ‘West-Med’ lineage in section Ilex as defined by Simeone et al. 2016)

**3 individuals**

[su23MO su24MO su25MO]

1. Based on all available data in gene banks (see also Simeone et al. 2016, fig. 1), these three *trnH-psbA* accessions are unrepresentative for their respective species. They differ markedly from other accessions of the same or putatively closely related species of section *Ilex*. The extent of mixing of *Cerris-, Cyclobalanopsis-* and *Ilex*-unique haplotypes in Asian species of sect. *Ilex* can so far not be accessed, and would require in-depth haplotyping across all species and their entire ranges. Nonetheless, currently, most western Asian *Ilex* accessions fall within the WAHEA lineage of sect. *Ilex*, whereas species thriving in East Asia increasingly show haplotypes closer related to sect. *Cyclobalanopsis.* *Cerris-*like haplotypes are rare; only *Q. phillyraeoides* consistently shows plastid signatures closely related to those typically found in sect. *Cerris* (cf. Simeone et al. 2016, fig. 1). [↑](#footnote-ref-1)
