# Supplementary figures and images for: Comparative systematics and phylogeography of Quercus Section Cerris in western Eurasia: inferences from plastid and nuclear DNA variation
Source: PeerJ. 2018 Oct 17;6:e5793. doi: 10.7717/peerj.5793 (PMC6195796; doi:10.7717/peerj.5793)

tioned sequence of *Quercus* section *Cerris*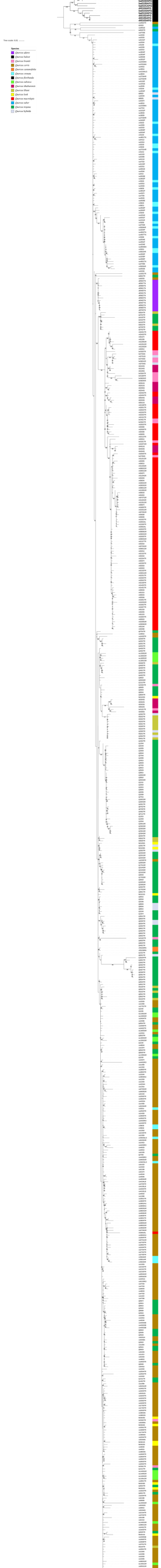

Supplement: File S4 — The tree was rooted on Q. baloot and Q. floribunda. Taxa colors as in Fig. 1, 2a and File S1. Branch bootstrap support (1-100) is reported. [file peerj-06-5793-s004.pdf]
