# Supplementary material for: Comparative systematics and phylogeography of Quercus Section Cerris in western Eurasia: inferences from plastid and nuclear DNA variation
Source: PeerJ. 2018 Oct 17;6:e5793. doi: 10.7717/peerj.5793 (PMC6195796; doi:10.7717/peerj.5793)

Supplemental File S5

Splitstree network based on PBC-transformed uncorrected distances of the 5S-IGS sequences. All individuals represented by ≥2 cloned sequences are included

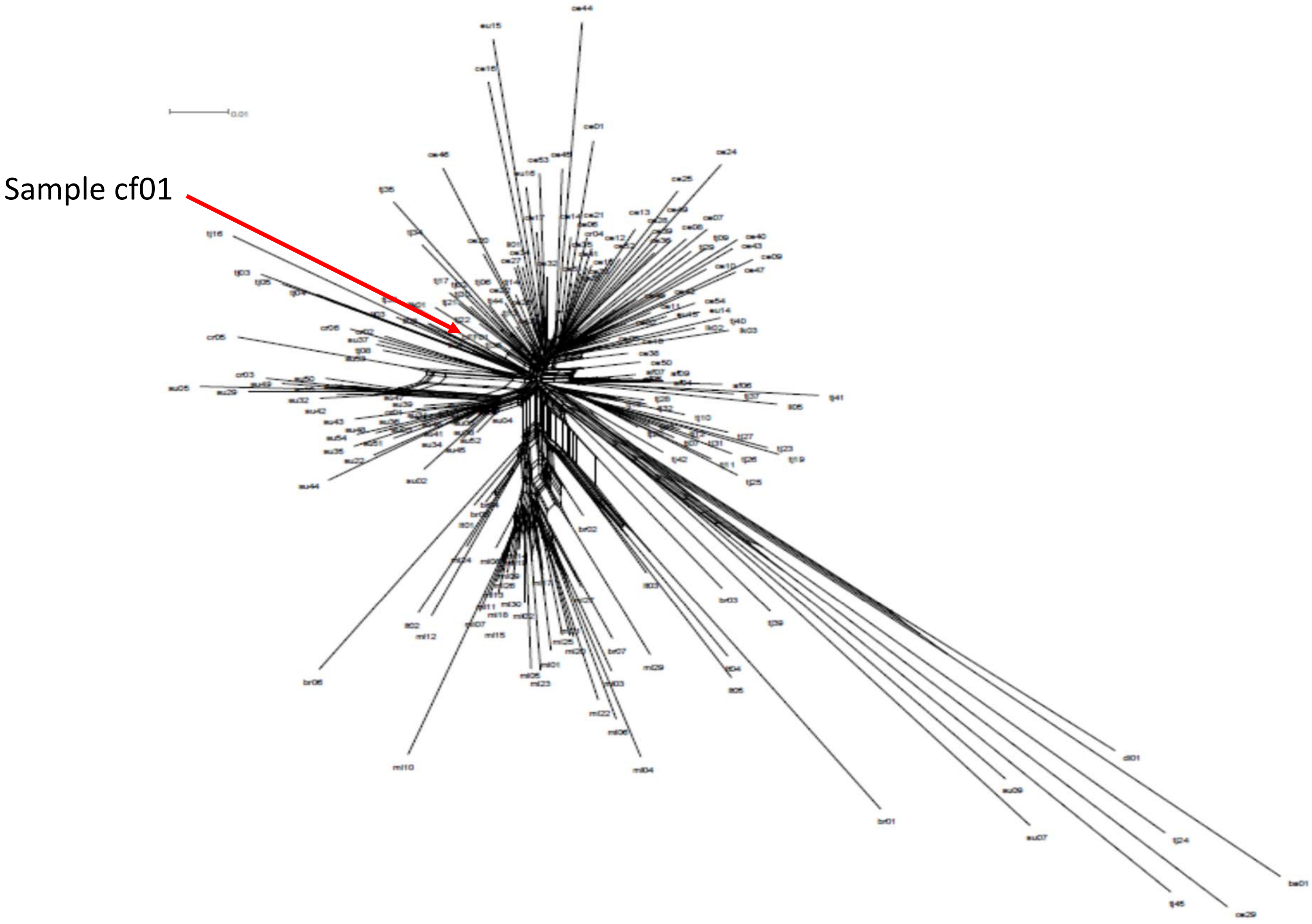

Supplemental File S5

Splitstree network based on PBC-transformed uncorrected distances of the 5S-IGS sequences. All individuals represented by ≥3 cloned sequences are included

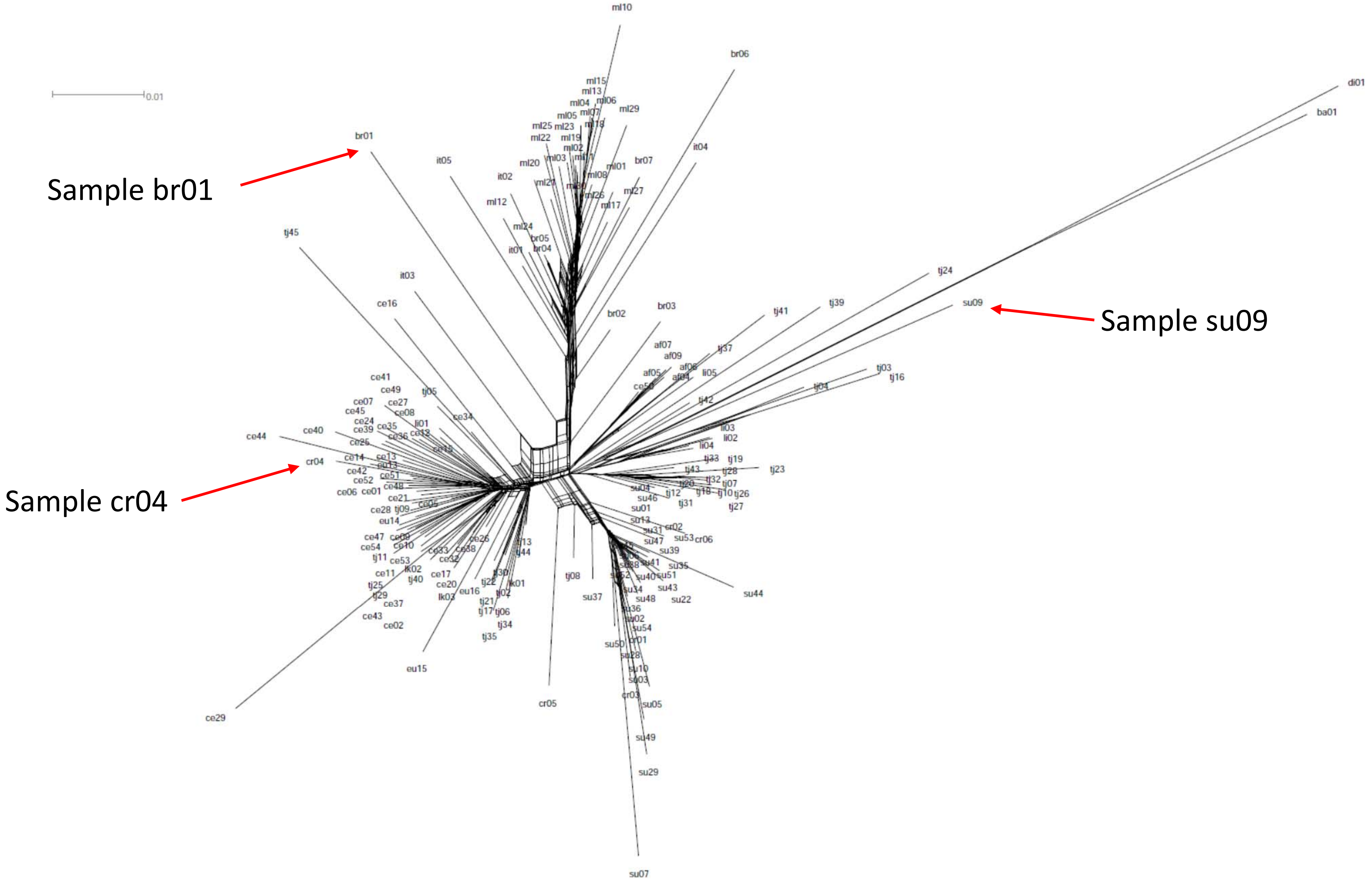

Supplemental File S5

Splitstree network based on PBC-transformed uncorrected distances of the 5S-IGS sequences. All individuals represented by ≥5 cloned sequences are included

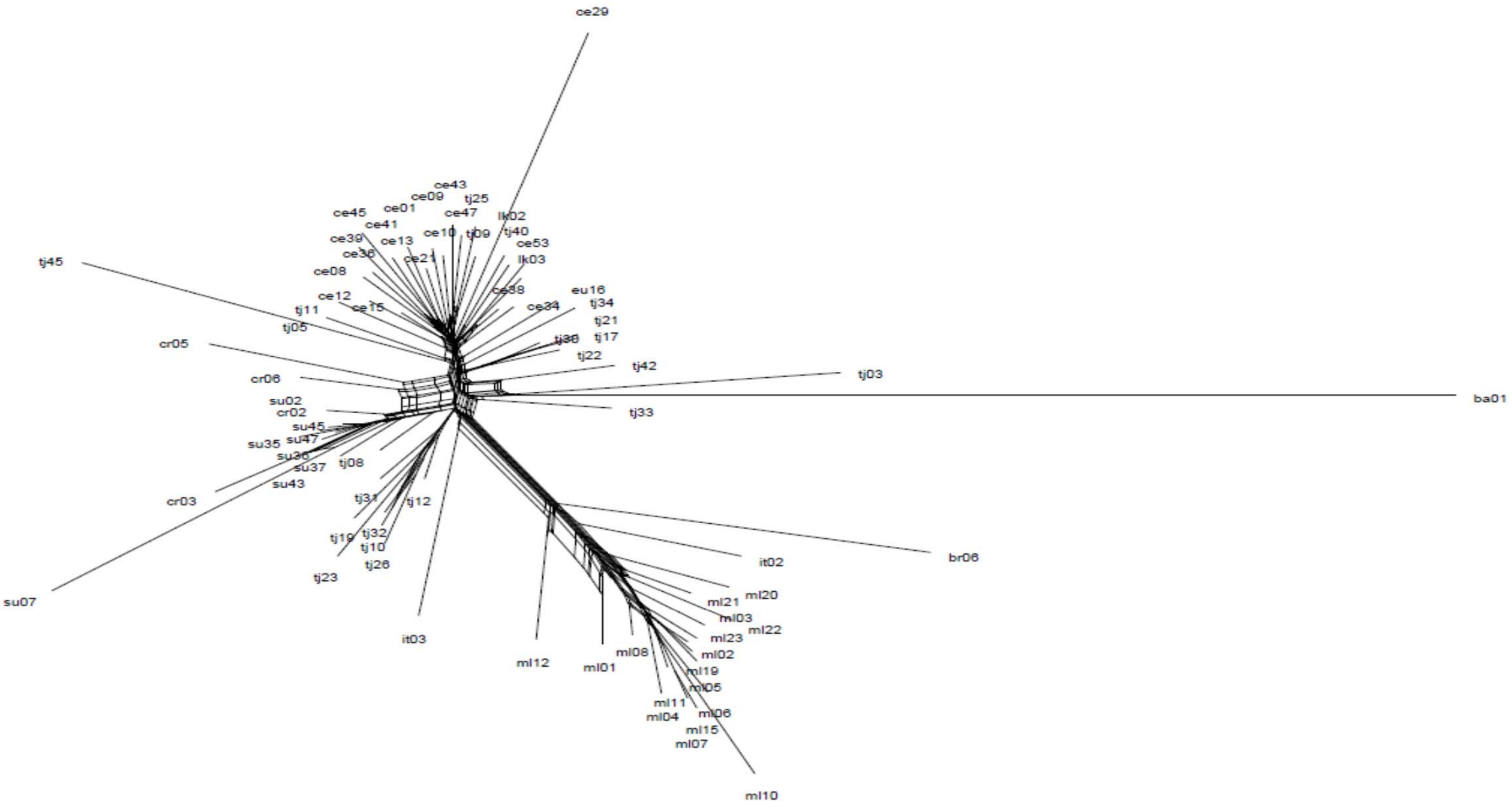

Supplement: File S5 — Individuals represented by more than 2, 3 and 5 clones are included (cut-offs specimens are indicated). [file peerj-06-5793-s005.pdf]
